# The changing face of *MedEdPublish*

**DOI:** 10.12688/mep.17500.1

**Published:** 2021-11-02

**Authors:** Richard Hays, Trevor Gibbs, Julie Hunt, Barbara Jennings, Ken Masters

**Affiliations:** 1 College of Medicine and Dentristy, James Cook University, Townsville, QLD, 4814, Australia; 2 AMEE, 12 Airlie Place,, Dundee, DD1 4HJ, UK, UK; 3 College of Veterinary Medicine, Lincoln Memorial University, Harrogate, Tennessee, TN 37752, USA; 4 Norwich Medical School, UNiversity of East Anglia, Norwich, Norfolk, NR4 7TJ, UK; 5 Medical Education and Informatics, Sultan Qaboos University, Al Khoudh, Oman

**Keywords:** medical education, publishing, open science

## The changing face of *MedEdPublish*

*MedEdPublish* began about 10 years ago as part of the Association for Medical Education in Europe (AMEE) *MedEdWorld* initiative, aiming to provide opportunities for members to share more formally their ideas, innovations and experiences in health professions education.

In 2016, *MedEdPublish* was re-launched as a separate e-journal using AMEE’s own publishing platform. This provided low-cost, open access publishing opportunities for a wide range of article types and a more direct relationship between authors, reviewers, and readers ( Hays, 2016). Editorial bias was reduced substantially because all of the articles were approved for publication if they met formatting requirements, had appropriate ethics approval and were within the scope of health professions education. To encourage constructive discussion threads, the review process was open and post-publication, with all reviews and comment published with the names of their contributors. Uniquely in health professions education, authors had an opportunity to submit a revised article based on reviewers’ comments. Finally, while all published articles remained accessible at no cost, those that received ‘recommended’ status, based on panel member reviews, were to be indexed in major publishing databases.

These innovations were so popular that the numbers of article submissions increased from 201 in 2016 to 552 in 2020, stretching the capacity of the AMEE in-house publishing resources. The resulting longer *submission to publication* times threatened our goal to be rapid and responsive to debates within our community of practice. Despite the challenges, the journal processed a total of 1758 articles during this time. About 80% of these have been published. All have received reviews, many sparking discussion threads that offer learning opportunities for authors, readers, and reviewers. Approximately 40% of all these articles have achieved ‘Recommended’ status. Re-submissions were received for about 5% of articles, with many improving their reviewer and reader ratings. 

October 2021 sees the launch of the next evolutionary phase of *MedEdPublish*, in partnership with Taylor and Francis, the publishers of AMEE’s well-established *Medical Teacher* journal as well as other international health professions education journals. The change has created an opportunity to use the F1000 platform, one which achieves a greater scale than the features that *MedEdPublish* pioneered. *MedEdPublish* is still the only open-access, post-publication peer review journal in the field of health professions education. In this article, we explain a little more about the changes and how they will improve the publishing experience.

## Additional features of *MedEdPublish* on the F1000 platform

The main advantage of the move to the F1000 platform is the technology that supports almost all of the innovative publishing features pioneered by *MedEdPublish* since 2016. These are summarised in Table 1.

**Table 1.** Features of the *MedEdPublish* F1000 platform.

There is capacity to process and store a higher volume of articles An *open data* policy will be followed for research articles reporting data Authors must nominate reviewers, although other potential reviewers may be invited Reviews are more focused on quality, particularly for research articles Reviews are also published, available with a DOI and citable The number of revisions and article updates is not limited Article processing fees have increased, although there are discounts for AMEE members and educators from resource constrained countries

The ‘back office’ functions are greatly enhanced, with increased use of semi-automatic decision software that can manage a much larger volume of articles within a rapid time frame and store a much larger number of articles. Articles in previously published volumes 5b-10 will migrate to the new platform, though additional reviews and revisions to these articles will no longer be permitted, although readers’ comments may be added.

The review process is a little different. Authors must nominate independent reviewers (who may or may not be contacted), and the software uses algorithms to select reviewers from both the author-nominated list and a pool of ‘registered reviewers’. Registered reviewers are approved by AMEE and acknowledged as having the necessary experience and expertise to meet F1000 requirements for recent activity in health professions education teaching, research and/or publishing. While these more eminent reviewers are important, particularly for research articles, we remain keen to attract more junior reviewers and support them in developing review expertise ( Hays *et al*., 2019). To maintain transparency, reviews will continue to be published, along with the name of the reviewers. All reviews are assigned a DOI and are themselves citable resources. The Advisory Board is available to nominate reviewers where the software cannot identify sufficient potential reviewers. 

*MedEdPublish* now aligns itself more closely with the principles of Open Science (incorporating Open Scholarship ( Burgelman *et al*., 2019)). Published articles must include citations to all repositories that host data, together with any software and code that was used for analyses. Open Science is standard practice for clinical, biomedical and humanities research publishing; there are well-documented advantages for both the research community ( Burgelman *et al*., 2019) and the author, including an increased citation rate ( Piwowar *et al*., 2007). For example, if other researchers can see raw data, replicate analyses, or pool individualised data through meta-analysis, it can result in rapid progress for some research fields ( Piwowar *et al*., 2011). For the author, transparent working practices tend to make research less error-prone and more efficient over time ( Ioannidis & Khoury, 2011). However, the challenge for using reproducible and open-data protocols is the necessary investment of time and other resources in data management systems, and ethics ( Burgelman *et al*., 2019; King, 2011). Looking towards the future of publishing, it will be interesting to see how extending this concept to health professions education publishing impacts on scholarly activities.

Openly sharing all data will require authors to ensure that the appropriate ethics approval and any required copyright releases have been obtained. In addition, because health professions education data are frequently about individuals, authors should take steps to anonymise raw data. Some ethical and data protection scenarios may mean that it is not feasible to openly share all data, in which case authors can contact the journal for guidance. A recent article covered ethical issues in publishing for health professions education ( Hays & Masters, 2020). Please note that this policy is relevant for all articles that report and analyse data, either quantitative or qualitative.

*MedEdPublish* will no longer limit the number of revisions or article updates. Authors may submit further revisions to address further feedback provided on earlier revisions. An interesting new feature is the *Update* feature, which is different from a *Revision*. The *Update* feature allows researchers to publish an update to their paper even after it has been accepted and indexed to share new information, changes in relevance and implications to the community of practice, and recent developments to the research published in the original paper. The increase in article processing fees reflects the real cost of publishing a high quality, innovative product and discounts will continue for AMEE members and authors from resource-constrained countries.

## What has not changed

Although *MedEdPublish* has entered a new and exciting phase of its development, the original ethos of rapidly publishing high quality post-publication, peer-reviewed articles remains at the core of all that we do. On its new platform, *MedEdPublish* will remain an AMEE journal with a focus on health professions education. The former Editorial Board will continue as the Advisory Board in order to provide continuity and oversight during this transition. *MedEdPublish* will continue to accept and publish a wide range of scholarly articles, including original research, teaching tips, opinion pieces, and conference reports. The publishing process will be rapid, transparent, and with minimal editorial bias, as articles meeting the journal’s criteria are uniformly accepted for publication.

We will continue to follow an *open identities* principle, which means all reviewers submit their feedback attached to their name, so that readers can see all peer-review reports, referee names, and comments linked to the article--and can join the discussion if they are registered users. Authors are encouraged to reply to reviews and comments in an open dialogue. We trust that this model will continue to enhance individual and community scholarship, with open discussion occurring between students and practising clinicians, between early-career and established researchers, and across professional specialties via the peer review process.

*MedEdPublish* will continue to make both original and revised versions available to readers, from now on kept all together and accessible through a single search. Articles receiving good ratings by selected reviewers will receive recommended status, which is linked with indexing of the article. As always, *MedEdPublish* articles are protected by Creative Commons licences, allowing broader dissemination and sharing. We believe that the additional features will greatly strengthen the journal and will continue to provide a useful platform for authors to showcase their work. These features are summarised in Table 2.

**Table 2.** What has not changed in the ‘new’ *MedEdPublish*.

*MedEdPublish* remains with AMEE as the ‘owner’ The former Editorial Board continues as the Advisory Board Our focus remains on health professions education We are keen to publish a wide range of scholarly articles, not just research The publishing process is rapid and transparent Editorial bias is reduced Reviews are post-publication and an ‘open identities’ principle is used Registered users can post comments on articles and reviews Authors are encouraged to respond to reviewers’ comments and submit revisions The original version, reviews and re-submissions are co-located as a continuous ‘live’ resource Articles that ‘pass’ peer review (are recommended by reviewers) are indexed in external databases Articles are published under Creative Commons (CC-BY) licence, as required by an increasing number of research grant organisations

## Accessing previously published *MedEdPublish* articles

Articles in Volumes 1-5a are available on the AMEE website. Volumes 5b-10 will migrate by the end of 2021 to the F1000 MedEdPublish website, although after October 2021 these can no longer be reviewed or revised.

## Register as a reviewer

The article processing software can prompt authors with names of reviewers with relevant expertise. Should you want to add your name to the list of potential reviewers, please contact *editorial@MedEdPublish.org*. There is an application form to complete, and a copy of your CV is required. People not on this list may also be contacted if the F1000 platform identifies them as subject matter experts.

## Please consider submitting your articles

Categories of articles that are welcome include research, brief reports, reviews, software tools, new educational methods, case studies, data notes, study protocols, practical tips, correspondence, and opinion articles. All topics in health professions education are welcome: curriculum development, assessment, quality assurance, identity formation and professionalism, the role of foundation sciences and humanities in education, clinical skills and simulation, and career and workforce development. Articles should be based on evidence, whether primary data or reviews with appropriate methods. Ideas, experiences and innovations should have potential for generalisation beyond the institution of the authors. Opinions should focus on solutions rather than problems and clearly describe how they were formed. Reports that ‘show negative’ results (no evidence of success of interventions) are welcome. Details how to submit are located at: https://submission. *MedEdPublish*.org/for-authors/publish-your-research/
